# Supplementary material for: A Homologous Recombination System to Generate Epitope-Tagged Target Genes in Chaetomium thermophilum: A Genetic Approach to Investigate Native Thermostable Proteins
Source: Int J Mol Sci. 2022 Mar 16;23(6):3198. doi: 10.3390/ijms23063198 (PMC8951082; doi:10.3390/ijms23063198)
Supplement: Supplementary file 1 [file ijms-23-03198-s001.zip › ijms-1621602-supplementary.pdf]

Supplementary Material

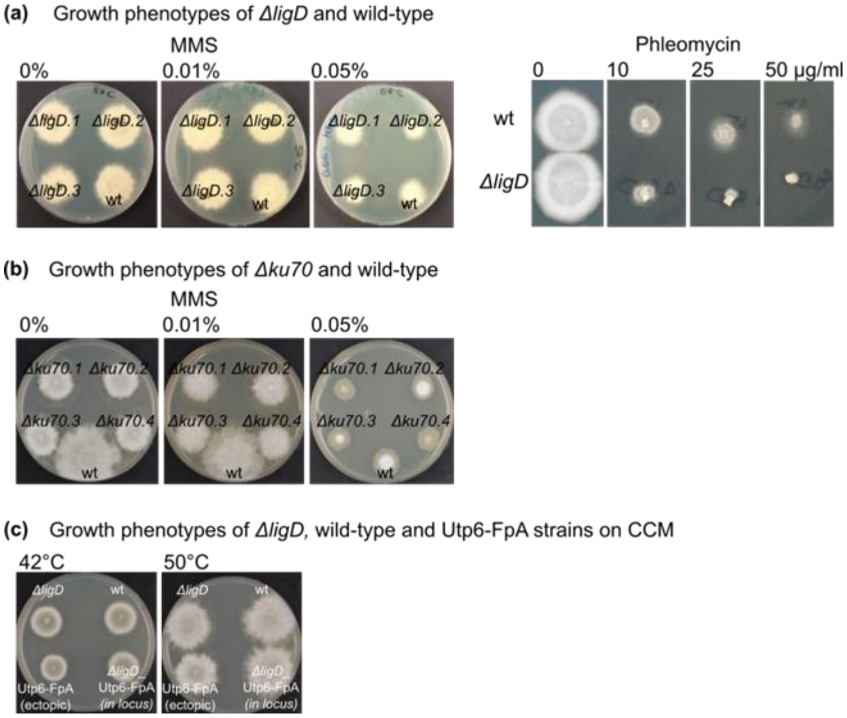

Figure S1. Growth phenotypes of the indicated strains. (**a**) Growth of three individual mycelia derived from single ascospores of strain *ΔligD#56* on CCM supplemented with DNA double strand break inducing agents, compared to the wild-type. The indicated concentration of either MMS (left panel) or phleomycin (right panel) was added to the medium. For growth on phleomycin only one clone is shown. Mycelial growth was documented upon incubation for 24 hour at 50 °C. (**b**) Growth of four individual mycelia derived from single ascospores of strain *Δku70#2* on CCM supplemented with the indicated concentration of MMS. (**c**) Growth phenotypes on CCM solid medium of the different Utp6-FpA-tagged strains in comparison to the *ΔligD* recipient strain and the wild-type at the indicated temperatures.
